# Extra-Mediterranean glacial refugia in a Mediterranean faunal element: the phylogeography of the chalk-hill blue *Polyommatus coridon* (Lepidoptera, Lycaenidae)

**DOI:** 10.1038/srep43533

**Published:** 2017-03-07

**Authors:** Gero Kühne, Joachim Kosuch, Axel Hochkirch, Thomas Schmitt

**Affiliations:** 1 Department of Biogeography, Faculty Regional and Environmental Sciences, Trier University, D - 54286 Trier, Germany; 2 Senckenberg German Entomological Institute, D - 15344 Müncheberg, Germany; 3 Zoology, Institute of Biology, Faculty Natural Sciences I, Martin Luther University Halle Wittenberg, D - 06099 Halle (Saale), Germany.

## Abstract

Most warm-adapted Central European species are thought to have survived ice ages exclusively in Mediterranean refugia. During recent years, this point of view has been questioned. Therefore, we tested the hypothesis that extra-Mediterranean refugia also played a role in warm-adapted insect species and selected the chalk-hill blue, *Polyommatus coridon*. We sequenced two mitochondrial loci (COI, CR) in 150 individuals from 30 populations covering nearly the complete range. Minimum spanning networks and other statistical analyses concordantly revealed four genetic lineages with strong phylogeographic signal: a western group in Italy, France and western/central Germany, an eastern lineage in the Balkan Peninsula, the Carpathian Basin and eastern Central Europe, an Alpine group with populations in the Alps and southern Germany and a Pyrenean group. Our results are generally consistent with previous analyses for *P. coridon* based on allozymes and DNA sequences, but provide additional insights. We propose that these four lineages have evolved during allopatry in different glacial refugia, two in typical Mediterranean refugia (Apennines and Balkan Peninsulas), but two in extra-Mediterranean areas south of the Alps and Pyrenees. This supports survival of warm-adapted organisms in these regions in close geographic proximity to the refugia of high mountain species.

The Pleistocene climatic oscillations had severe impacts on the distributions of many animal and plant species^1^,^2^,^3^. However, the influence of climatic changes on distributions strongly varies among species due to their diverse ecological requirements. In temperate regions, the geographic ranges of warm-adapted taxa expanded polewards during warm periods and retracted towards the equator when the climate became cooler. By contrast, cold-adapted species expanded their ranges from mountain and arctic refugia during cold periods^4^,^5^. These cyclic range shifts resulted in characteristic biogeographic patterns in different regions of the world^2^. In Europe, three main biogeographic groups (i.e. faunal elements) represent the large majority of extant animal and plant species: the Mediterranean, the continental and the arctic/alpine type^6^.

It has long been proposed that species belonging to the Mediterranean faunal element survived in refugia situated around the Mediterranean Sea during the glacial phases of the Pleistocene^7^,^8^,^9^. This hypothesis has meanwhile been supported by numerous genetic studies (reviewed in Hewitt^2^). Additionally, the polycentricity of the larger refugia (i.e. Iberia, the Apennines Peninsula, the Balkans, the Maghreb and Asia Minor) has repeatedly been demonstrated (reviewed in Schmitt^6^; Husemann *et al*.^10^). The hypotheses of postglacial range expansions from these Mediterranean refugia have largely been confirmed, and extensive studies of the distribution of genetic variability in the western Palaearctic revealed four paradigms of postglacial range expansion in Europe^11^,^12^.

The group of continental species includes the majority of taxa that previously have been assumed to be Siberian or Manchurian elements. For these species, postglacial expansion from Central or East Asia to Europe had been postulated^13^. However, this assumption has been discussed controversially since the 1970ies (for details see Schmitt & Varga^14^), and the possibility of glacial survival in European extra-Mediterranean refugia has been proposed^15^. The existence of such retreats in Europe has been demonstrated by genetic analyses for a variety of taxa over the last two decades (reviewed in Schmitt & Varga^14^).

The main process formerly discussed for arctic-alpine and alpine disjunct species is a massive spread during the cold stages of the Pleistocene leading to large continental distributions, followed by inter- and postglacial retreat to high mountain systems or the Arctic realm^4^,^16^. This point of view has largely been confirmed for arctic-alpine species, but the situation seems to be more complex in purely alpine species (often with disjunct distributions over several high mountain systems). These species most probably have survived glaciations in refugia at the foothills and lower parts of high mountains (e.g. Alps, Pyrenees or Carpathians) or in the hilly areas between such high mountain systems, but not in large continental regions (reviewed in Schmitt^17^).

However, there is accumulating evidence that the repetitive patterns in these three biogeographical groups are not as paradigmatic as previously thought. Particularly the paradigm of exclusive Mediterranean refugia for “Mediterranean” taxa has recently been questioned^3^,^18^. Pollen analyses confirmed glacial survival of a number of cold-tolerating tree species in the Carpathians^19^,^20^,^21^,^22^,^23^, and genetic analyses proved Würm ice age refugia of the Common Beech, *Fagus sylvatica*, in several extra-Mediterranean regions^24^. Similar examples exist for animal species: The Brown Bear, *Ursus arctos*, once being selected for the bear paradigm of “Mediterranean” species, had extra-Mediterranean occurrences during the last ice age at least in the Carpathians^25^,^26^. Carpathian ice age refugia have also been demonstrated for *Bombina* toads^27^. Endemic haplotypes of the Common Vole, *Microtus arvalis*, in the area of the Black Forest in Germany imply glacial survival in the vicinity of these mountains^28^. Further extra-Mediterranean ice age refugia were demonstrated for otherwise Mediterranean faunal elements, such as the newts *Ichthyosaura alpestris*^29^ and *Triturus vulgaris*^30^.

In Italy, apart from numerous known core areas in the southern part of the country, some amphibians and reptiles had their ice age refugia in the Plain of the Po^31^,^32^,^33^,^34^, i.e. at the extreme northern edge of the Adriatic-Mediterranean centre or even in the extra-Mediterranean part of Europe. However, the importance of peri-Alpine areas as retreats for warm-adapted species, i.e. in close geographic proximity to high mountain species, is mostly unknown, and so far, apart from the butterfly *Euphydryas aurinia*^35^, no example exists for invertebrate peri-Alpine refugia.

We hypothesized that warm-adapted species, i.e. taxa that in Europe have to retreat southwards during glacial periods, also survived at least the last ice age in extra-Mediterranean refugia in close proximity to high mountain systems. We selected the chalk-hill blue, *Polyommatus coridon* (Poda 1761), a common lycaenid butterfly species of semi-natural calcareous grasslands^36^,^37^,^38^,^39^, as an example. This species is widespread in South and Central Europe^40^. The butterflies are sedentary and only some cases of long distance dispersal have been documented^37^,^38^,^41^. In western Europe, the larvae exclusively feed on *Hippocrepis comosa*^37^, but eastern populations frequently use *Securigera varia*^42^. *P. coridon* is mutualistically associated with several ant species, a common phenomenon of lycaenid butterflies^43^,^44^.

Allozyme studies of *P. coridon* revealed two major evolutionary lineages: a western lineage in Italy, France and Germany (representing the Adriatic-Mediterranean element), and an eastern lineage in eastern and south-eastern Europe (Pontic-Mediterranean element)^45^,^46^. These two lineages were also supported by mtDNA analyses^47^. The genetic diversity decreases towards areas colonised during the postglacial period, probably due to repeated bottlenecks during the process of expansion^48^,^49^. The western allozyme lineage showed some genetic differentiation into four groups of populations: Pyrenees; Central Italy; France, western and central Germany; southern Germany^49^,^50^. Allozyme markers also revealed a clear differentiation of *Polyommatus hispana* (Herrich-Schäffer 1852) from *P. coridon*, supporting species status of both taxa^46^. However, Talavera *et al*.^47^ could not discriminate between these two species applying mitochondrial and nuclear DNA sequences.

Today, the chalk-hill blue is also found at higher altitudes in mountains^51^,^52^, thus suggesting survival north of the classical Mediterranean refugia. To test this assumption, we sequenced two fragments of the mitochondrial genome (COI and Control Region) of individuals from thirty *P. coridon* populations covering almost the entire distribution of this species.

## Material and Methods

### Sampling design

**Figure 1:**
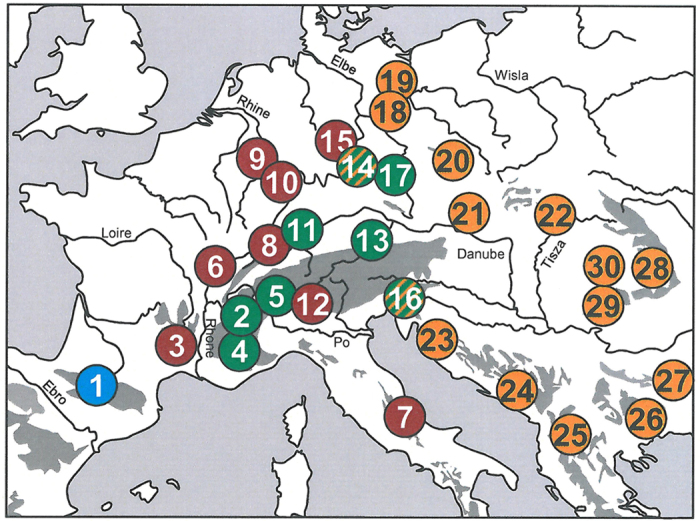
The thirty sampling localities of *Polyommatus coridon* are scattered over most of the species’ extant distribution range. All numbers refer to Table 1. Colours indicate the four different haplotype groups referring Fig. 2. Striped localities have haplotypes belonging to two different groups. Map was generated with Microsoft PowerPoint 2010.

Butterflies of 30 *P. coridon* populations were netted across Europe from 1996 to 2004 (Fig. 1, Table 1). All these populations (except for Panticosa, Spanish Pyrenees) have previously been studied by allozyme electrophoresis^45^,^46^,^48^,^49^,^53^; including additional unpublished data). Samples were stored in liquid nitrogen immediately upon capture. As outgroups, we added five individuals of *P. hispana* (La Braisse, F) and one *P. icarus* (Trier, D).

### Sequencing of mtDNA

We examined variation in two mtDNA fragments for five specimens per locality. DNA was extracted from the head tissue using the DNeasy Blood & Tissue Kit (Qiagen GmbH, Hilden, Germany) following the manufacturer’s protocols. We amplified two mtDNA gene fragments, the Cytochrome Oxidase I (COI) and the Control Region (CR). PCR amplifications were performed with PuReTaq Ready-To-Go PCR beads (GE Healthcare, Little Chalfont, UK). We added 4 μl DNA extract, 0.2 μl of each primer (100 pmol/μl) and 15.6 μl water to each reaction tube. The primers k698 TY-J-1460 (TAC AAT TTA TCG CCT AAA CTT CAG CC) and Nancy C1-N-2192 (GGT AAA ATT AAA ATA TAA ACT TC)^54^ were used to amplify a 713 bp fragment of the second half of the COI gene; the primers LepAT2B (ATT AAA TTT TTG TAT AAC CGC AAC) and SeqLepMet (TGA GGT ATG ARC CCA AAA GC)^55^ were used for the amplification of a 548–556 bp fragment (after removing a microsatellite sequence) of the CR gene.

The PCR program for COI started with denaturation at 94 °C for 4 min, 35 cycles of denaturation at 94 °C for 30 s, annealing at 41 °C for 30 s and an extension step at 72 °C for 60 s. The final extension step took 4 min. For the CR gene, the reaction started with a first denaturation step at 94 °C for 2 min, followed by 35 cycles of denaturation at 94 °C for 60 s, annealing at 54 °C for 60 s and extension at 65 °C for 60 s. The last extension step took 11 min.

PCR products were run on a 1.4% agarose gel, stained with ethidium bromide and checked visually under UV light. Positive PCR products were purified with the QIAquick PCR Purification Kit (Qiagen GmbH, Hilden, Germany) and used for single stranded sequencing with the primer k698 TY-J-1460 for COI and LepAT2B for CR. Sequencing reactions and sequencing were performed by Seqlab GmbH (Göttingen, Germany). All sequences were aligned with the Sequence Navigator software (Applied Biosystems, Lincoln, CA, USA)^56^ and subsequently refined by eye using Mega 4.0^57^. Sequences were deposited in GenBank under the accession numbers KR007004-KR007307.

### Data analysis

Unrooted minimum spanning haplotype networks based on statistical parsimony were calculated for each gene fragment using Tcs 1.18^58^; default settings. Reticulations were resolved following Pfenninger & Posada^59^. To determine the potential root of the network, we included the outgroups *Polyommatus hispana* and *Polyommatus icarus* in a maximum parsimony (MP) analysis. We calculated an MP tree with Paup 4.0b10*^60^ and used a heuristic search (TBR branch swapping), treating gaps as fifth character state. Confidence of the nodes was evaluated by bootstrapping the matrix 10,000 times^61^. Furthermore, we inferred the phylogeny in MrBayes 3.2.6^62^. We first used PartitionFinder^63^ to determine the best fitting substitution model using the greedy algorithm. For this, we divided the data set into four partitions: the control region and the three codon positions of the COI data set. Based upon AIC, we selected the following models with linked branch length for the Bayesian analysis: Control region and CO1 codon position 2: GTR + I + G, COI codon position 1: GTR + I, COI codon position 3: HKY + G. In MrBayes, we ran two runs with 10 million generations and four independent chains (one cold and three heated chains) and sampled every 1000^th^ generation. *Polyommatus icarus* was chosen as outgroup. A majority rule consensus tree was built after a burn-in of 20%.

Based on the concatenated mtDNA data set, we grouped populations based on haplotype distributions by applying a spatial analysis of molecular variance (SAMOVA 1.0)^64^. This approach maximises the proportion of explained total genetic variance due to differences between groups of populations for any predefined number of groups. We increased the number of groups as long as the resulting geographic structure of separated populations showed a coherent spatial distribution. Based on this grouping, we performed an AMOVA^65^ as implemented in SAMOVA 1.0 to evaluate the genetic variance explained within and among these population groups. Significance of variance components was determined after 1,023 permutations.

For each of the population groups revealed by SAMOVA, we estimated nucleotide diversity *Π*^66^, raggedness statistic *r*^67^ and Ramos-Onsins & Rozas’^68^ *R*^2^. The latter quantifies the smoothness of the observed pairwise differences distribution (all calculations with DnaSP version 4.10.9^69^.

The mismatch distribution of populations (calculated with DnaSP^67^, fused to regional population groups (see above), is shaped by the population history. We therefore compared the observed mismatch distribution to two models of population development: continuous growth model *versus* expansion and decline model. Since observed mismatch distributions may be caused by more than one population process^70^, we carefully interpret them only as additional evidence for otherwise inferred population histories.

## Results

We found 38 haplotypes for COI (150 individuals) and 76 haplotypes for CR (147 individuals). In case of CR, we excluded a microsatellite motive with a varying number of motive repetitions from the analysis. These diversity differences of the two gene fragments are in line with the higher substitution rate of CR, which is the only non-coding region of the mitochondrial genome in insects^71^. The maximum *p*-distance among *P. coridon* haplotypes was 0.022 for CR and 0.018 for COI. The five individuals of *P. hispana* had three COI haplotypes closely related with the *P. coridon* haplotypes observed in the Alps and southern Germany; the four CR haplotypes of *P. hispana* were shared with *P. coridon* in two cases and the remaining haplotypes were only distinguished by one and two substitutions, respectively. The haplotype of *P. icarus* differed strongly from the above mentioned species for both loci with mean *p*-distances of 0.061 to *P. coridon* and 0.059 to *P. hispana* for COI and 0.056 to both other species for CR.

**Figure 2:**
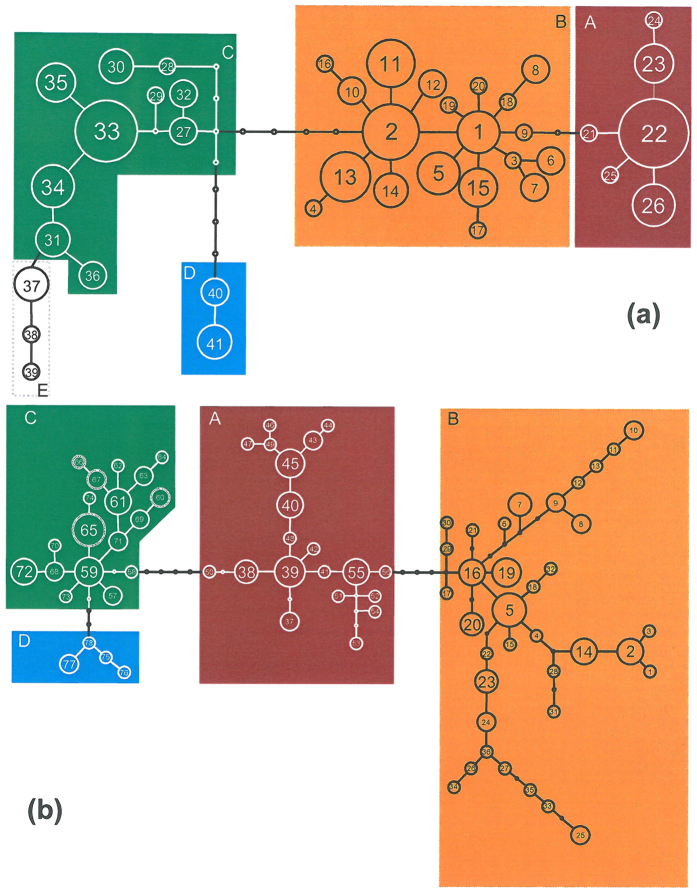
Haplotype networks of the mtDNA loci COI (**a**) and CR (**b**) of *Polyommatus coridon*. Four genetic lineages (A–D) were distinguished for *P. coridon*. Lineage E represents the sibling species *Polyommatus hispana*. For CR, no discrete genetic lineage existed for *P. hispana*, but its haplotypes were included in the *P. coridon* lineage C (haplotypes 65, 66 and 67, dotted). Lineage A (red) represents the western, B (orange) the eastern, C (green) the Alpine and D (blue) the Pyrenean lineage.

The most common COI haplotypes were represented by H22 (18.7%), H33 (11.3%), H02 (8.7%) and H13 (5.3%); all other haplotypes had overall frequencies of less than 5%. For the CR locus, none of the haplotypes showed such a high frequency as for COI, and the most common haplotype H05 had an overall frequency of only 5.4% (Fig. 2).

**Figure 3:**
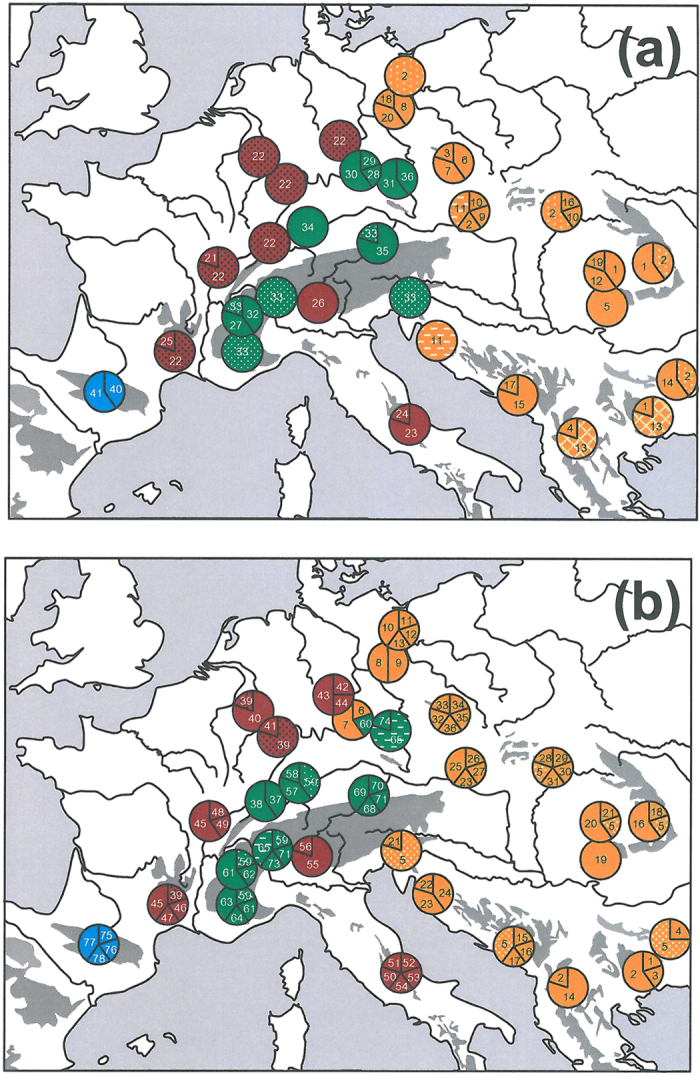
Geographic distribution of haplotypes of the mtDNA COI (**a**) and CR (**b**). All haplotypes are coloured referring their haplotype group (confer Fig. 2). The most common haplotypes have additional signatures in their lineage colour. Maps were generated with Microsoft PowerPoint 2010.

**Figure 4:**
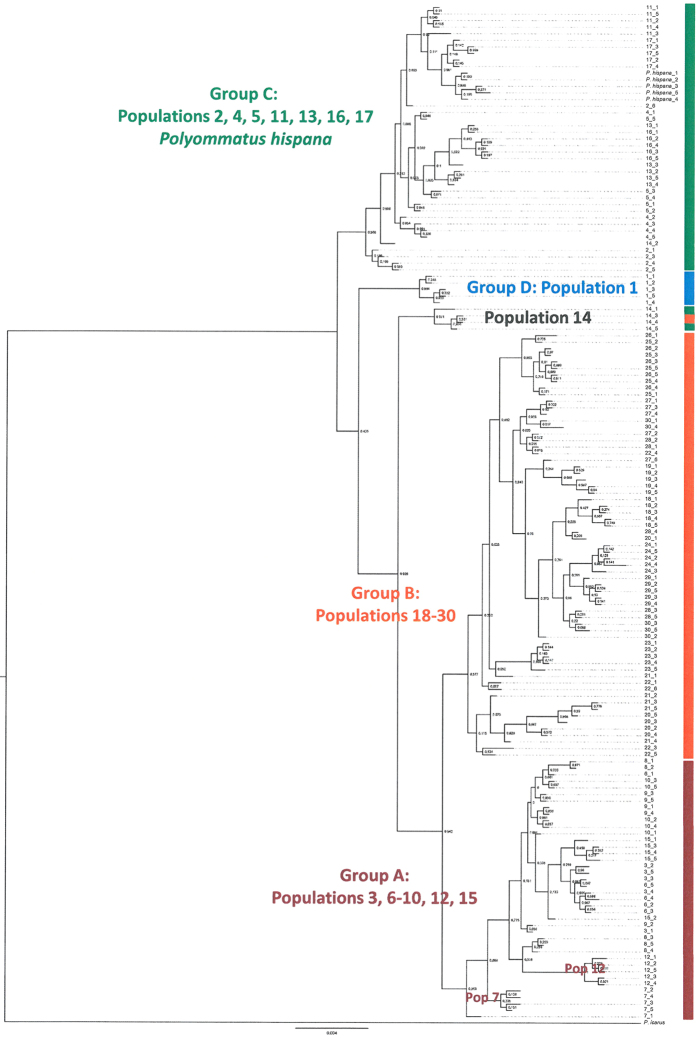
Bayesian phylogeny based on concatenated mtDNA (COI, CR) of thirty populations of *Polyommatus coridon* and one population of *P. hispana*. Partition Finder^63^ was used to determine the best fitting substitution model using the greedy algorithm. In MrBayes^62^, we ran two runs with 10 million generations and four independent chains (one cold and three heated chains) and sampled every 1000^th^ generation. *Polyommatus icarus* was chosen as outgroup. A majority rule consensus tree was built after a burn-in of 20%. Colours indicate the four different haplotype groups referring Fig. 2.

In both networks, we obtained four genetic groups with almost all populations having similar assignments in both cases: (A) a western European group (Italy, France, western Germany and one population from western Bohemia); (B) an eastern European group (East and Southeast Europe to central Bohemia and NE Germany); (C) an Alpine group (Alps and southern Germany) and (D) a Pyrenean group (Pyrenees) (Figs 2 and 3). These groups were separated by one to four substitutions in COI and three to four substitutions in CR. Only two populations (Tiefenstürmig in Bavaria; Hochobir in SE Austria) contained haplotypes assigned to different phylogroups. The root of the two haplotype networks determined by MP trees and hierarchical outgroups was located between the Pyrenean haplotypes and all others. The same structure is confirmed in the consensus tree from the Bayesian inference (Fig. 4).

No further phylogeographic structure was obtained within the eastern lineage B. Only in the CR locus, the haplotypes of Brandenburg (H8–H13) and Czech Republic were differentiated from the main body of the network. The haplotypes H25–H27 and H33–H36 from Czech Republic were linked by the three Croatian haplotypes 22–24 (population 23) to the main body. While no geographic structure was observed for the Alpine group C, some geographic structure occurred in the western group A: For COI, population 7 from Central Italy had the two exclusive haplotypes H23 and H24 distinguished by one and two substitutions, respectively, from the main haplotype of the group H22, which was rather widespread in France and Germany. For CR, four of the five different haplotypes of the central Italian group (H51–H54; i.e. a singleton for each individual of the group) clustered at a separate branch within this group. These haplotypes were linked via the haplotype H55 detected in Ticino (population 12) with the bulk of haplotypes from Germany and France, without any further geographic differentiation.

**Figure 5:**
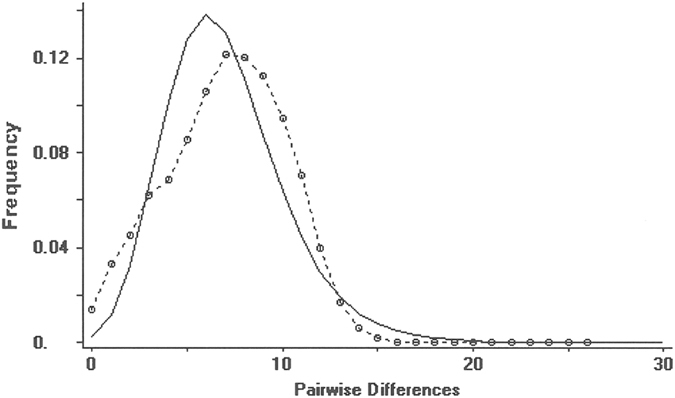
Observed (broken line) and expected (solid line) mismatch distribution under a growth-decline model for the complete data set of Control Region sequences of *Polyommatus coridon*.

Nucleotide diversity *Π* was highest for the haplotype groups A and B and lowest for group D, while raggedness was more pronounced in group D (Table 2). Mean *θ* values were high in population groups A, B and C and low for D. Mismatch distributions of the complete data set and single population groups show a pattern of population growth and decline (Fig. 5).

SAMOVA classified the populations into four groups. The grouping was nearly identical with the groups identified in the haplotype networks (Table 1). However, the Tiefenstürmig population (14) was grouped with the eastern group B, while it contains haplotypes of groups B and C. Only one population was grouped differently from the groupings found in the haplotype networks: Colla (Tissino, CH; population 12) was sorted into the eastern group B by SAMOVA, but to the western group A in the haplotype networks. Due to these assignment uncertainties, it was not possible to reasonably group the populations Tiefenstürmig and Colla for the hierarchical variance analysis. Therefore, these two populations were excluded in the following. We obtained a total genetic variance for the remaining *P. coridon* samples of 8.62. The four geographic groups explained 61.1% of the variance (*p* < 0.001), whereas 26.6% (*p* < 0.001) was explained by populations and 12.3% within populations. The inclusion of the populations Tiefenstürmig and Colla in their SAMOVA groups did not change this result remarkably. The overall *F*_ST_ was 0.877 (*p* < 0.001).

## Discussion

### Genetic structures

The pattern revealed by mitochondrial DNA is congruent with the pattern found in previous allozyme studies^45^,^46^,^48^,^49^ and the geographic distribution of chromosome numbers^70^, but shows a considerably higher resolution. The allozyme and chromosome analyses revealed only two major lineages, of which the eastern one is identical in its geographic extent to the eastern mtDNA lineage B, while the other three mtDNA lineages resemble a western group found in the allozyme^45^ and chromosome^72^ studies. Also the study presented by Talavera and colleagues^49^ based on mtDNA and nDNA sequencing detected differentiation between eastern and western *P. coridon*, but the geographic coverage of sampling was not sufficient to detect the four groups obtained in our study. However, substructures in the western allozyme group already resembled the differentiation pattern of our mtDNA sequences, but still were less clear-cut. Thus, the Pyrenean sample also for allozymes showed the highest genetic differentiation from all the other samples; samples from southern Germany represented a monophyletic group within the other samples from Germany, France and Italy, the latter hence being paraphyletic^49^. Thus, the three mtDNA lineages A, C and D are at least partly supported by structures previously observed in allozyme polymorphisms. This congruence between mtDNA and the nuclear marker allozymes let us argue that a biogeographic interpretation of our mtDNA pattern is not spoiled by effects of introgression, incomplete lineage sorting or *Wolbachia* infection that all are known to frequently imperil proper phylogeographic analyses.

### Glacial refugia and postglacial range expansion

Following the above approved assumption of an unspoiled phylogeographic pattern, the differentiation of *P. coridon* into four well distinguished genetic groups indicates four allopatric differentiation centres. As nowadays the species has a continuous distribution over major parts of Central and South Europe^40^, allopatry most likely occurred during the cold phases of the Pleistocene. While the differentiation between all groups covered just a few substitutions (one to five in COI and three to four in CR) and our data implies up to six substitutions in the CR during the postglacial period (see below), the onset of the entire differentiation in *P. coridon* apparently is rather recent and the application of a molecular clock might yield misleading results and therefore was not used. However, due to the obtained genetic structure, we assume glacial isolation during the Würm as the most likely trigger for this differentiation; a similar suggestion was made based on an extensive data set of allozyme polymorphisms^45^. However, even if the origin of differentiation in *P. coridon* is older than the last ice age (maybe starting already in Mindel or Riss), the geographic sorting of lineages should reflect the refugial situation during the Würm ice age and is discussed in the following.

#### Mediterranean refugia

The two mtDNA lineages A and B represent the classical Mediterranean refuge areas in the Adriatic- and in the Pontic-Mediterreanean region, respectively, as already postulated by de Lattin^9^. The intra-lineage differentiation of the western group A into haplotypes from central Italy (Gran Sasso), probably the Ticino and all others found in France and Germany supports a structured distribution during the Würm ice age. This genetic structure may even indicate a subdivision into several subrefugia scattered over the Adriatic-Mediterranean region. Phylogeographic structures for a variety of other species affirm this assumption, often with considerably stronger regional differentiation within Italy and thus numerous glacial refugia^31^,^32^,^33^,^34^,^73^,^74^.

**Figure 6:**
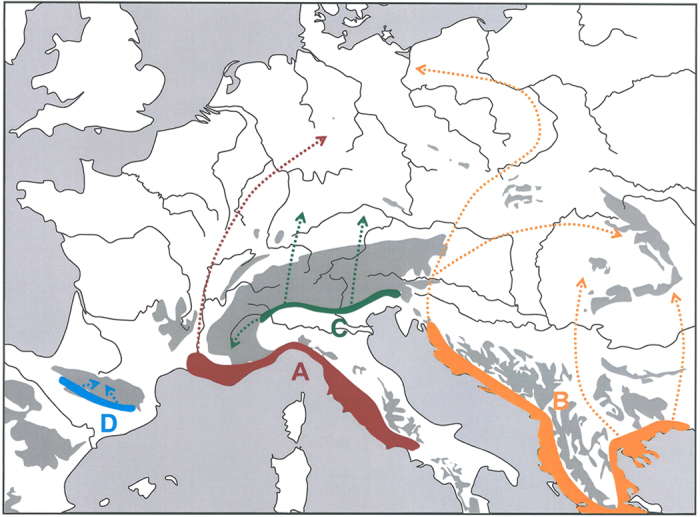
The analyses of DNA sequences of the two loci COI and CR support four Würm glacial refugia for the butterfly *Polyommatus coridon*: Adriato-Mediterranean, Ponto-Mediterranean, southern Alpine and Pyrenean. The refugia A (red) and B (orange) represent the Mediterranean refugia detected in previous publications. The refugia C (green) and D (blue) are additional extra-Mediterranean refugia. From the refugia A, B and C, major parts of Central and West Europe were colonised during the Postglacial (dotted arrows in the respective colours). Only the Pyrenean refugium D contributed little to the postglacial range expansion. Map was generated with Microsoft PowerPoint 2010.

*P. coridon*’s Adriatic-Mediterranean refugium with its putative subcentres most probably extended from central/southern Italy to SE France (Fig. 6). This latter region might have served as the leading edge during postglacial range expansion to most other regions of France, western and central Germany. Independent glacial refugia in the Provence also have been reported for other Mediterranean species^24^,^34^,^35^,^75^,^76^,^77^, thus making glacial survival of the chalk-hill blue in this region likely. The genetic homogeneity in the northern part of the range of this lineage (especially for COI, Fig. 3a) might be due to genetic erosion caused by founder events during the expansion process^78^.

This scenario is well supported by allozyme polymorphisms^49^. They show the strongest genetic differentiation within this group for the population from Gran Sasso, reveal a stronger genetic differentiation among populations within the postulated refugium than in the northern parts of the range and show no geographically meaningful differentiation over the entire postglacial expansion area, but genetic impoverishment of the more northern populations of this mtDNA lineage.

The eastern lineage B had the greatest haplotype diversity, but no meaningful phylogeographic substructure over most of south-eastern Europe. This might be explained by a more or less continuous glacial distribution of this lineage along the coasts of the Balkan Peninsula, with at maximum some local geographic discontinuities (Fig. 6). Therefore, a strongly allopatric glacial distribution with geographically distinguished ranges of the respective lineages, as observed in several other Mediterranean taxa in this region^34^,^73^,^79^,^80^,^81^,^82^,^83^, is little likely for *P. coridon*.

The population from Croatia was the only south-eastern European population included in a separate branch in the CR network; this branch otherwise was mostly found in the northern part of the lineage’s distribution. This supports the assumption that the north-western leading edge of the Pontic-Mediterranean refugium of *P. coridon* was located in the north-western Balkan Peninsula during the last cold stage. The most differentiated CR haplotypes in the group B were found in Brandenburg and Czech Republic, both of which form separate haplotype clusters with up to five and six substitutions between haplotypes, respectively. The Czech haplotypes most probably derive directly from the Croatian ones supporting postglacial expansion from the western Balkan Peninsula to Bohemia, as also supported by allozyme data^48^ and rapid evolution of different CR haplotypes during little more than 10,000 years. This is further underlined by exclusive CR haplotypes in Brandenburg, an area which was mostly covered by ice during the last glaciation^84^. Such a quick evolution is feasible to occur in the non-coding CR region and also has been observed in the clouded apollo, *Parnassius mnemosyne*^85^.

#### Extra-Mediterranean refugia

The remaining two groups C and D do not match the classical theory of the Mediterranean refugia^4^. Lineage D was exclusively composed of the only analysed Pyrenean sample. As the typical Mediterranean retreat areas of Iberia should have been blocked by the two sibling species *P. hispana* and *P. albicans*, the most likely evolutionary centre of this lineage is south of the Pyrenees and thus in the extra-Mediterranean part of Iberia and hence Europe.

The lineage C comprises populations from the Alps and southern Germany. This lineage probably did not survive the last ice age in one of the classical Mediterranean refugia; all of them were blocked by other lineages of *P. coridon* or are geographically separated from the recent distribution of this lineage. Therefore, this lineage most probably has survived the last glaciation in an extended refugium along the lower elevations of the southern Alps. This area is well known as refugium for many Alpine species (reviewed in Schönswetter *et al*.^86^ and Schmitt^17^) and also of some continental species (reviewed in Schmitt and Varga^14^). Furthermore, survival of a temperate plant, the beech *Fagus sylvatica*, was demonstrated in this area^24^. However, we demonstrate for the first time that this area was an important glacial refugium for a warm-adapted animal species. Consequently, representatives of all major biogeographic groups might have co-occurred in close geographic proximity at the southern slopes of the Italian Alps during, at least, the last ice age.

## Additional Information

**How to cite this article**: Kühne, G. *et al*. Extra-Mediterranean glacial refugia in a Mediterranean faunal element: the phylogeography of the chalk-hill blue *Polyommatus coridon* (Lepidoptera, Lycaenidae). *Sci. Rep.* **7**, 43533; doi: 10.1038/srep43533 (2017).

**Publisher's note:** Springer Nature remains neutral with regard to jurisdictional claims in published maps and institutional affiliations.

## Figures and Tables

**Table 1:** Number, name, country, geographic location, haplotypes (COI and CR haplotypes separated by semicolon) and SAMOVA assignment of all sample localities of *Polyommatus coridon*.

No.	Locality, country	Geographic coordinates	Haplotypes (COI; CR)	SAMOVA group
1	Panticosa, E	42°43′N 00°16′W	40,41; 75–78	D
2	Vallee des Glaciers, F	45°44′N 06°46′E	27,32,33; 59,61,62	C
3	Languière, F	44°04′N 03°07′E	22,25; 39,45–47	A
4	La Palud, F	43°52′N 06°30′E	33; 59,61,63,64	C
5	Täschalp, CH	46°03′N 07°49′E	33; 59,65,71,73	C
6	Velars, F	45°57′N 05°58′E	21,22; 45,48,49	A
7	Gran Sasso, I	42°26′N 13°31′E	23,24; 50–54	A
8	Griesheim, D	47°51′N 07°35′E	22; 37,38	A
9	Niederehe, D	50°18′N 06°48′E	22; 39,40	A
10	Bad Münster am Stein, D	49°50′N 07°53′E	22; 39,41	A
11	Dapfen, D	48°21′N 09°30′E	34; 57–59	C
12	Colla, CH	46°28′N 08°14′E	26; 55,56	B
13	Heutal bei Unken, A	47°40′N 12°42′E	33,35; 67,68,70,71	C
14	Tiefenstürmig, D	49°52′N 10°53′E	28,30,33; 6,7,60	B
15	Craula, D	51°09′N 10°35′E	22; 41–43	A
16	Hochobir, A	46°30′N 14°19′E	33; 5,21	C
17	Černín, CZ	49°56′N 13°59′E	31,36; 65,74	C
18	Pätz, D	52°15′N 13°41′E	8,18,20; 8,9	B
19	Gartz, D	53°08′N 14°20′E	2; 10–13	B
20	Milovice, CZ	50°12′N 14°56′E	3,6,7; 32–36	B
21	Klentnice, CZ	48°49′N 16°38′E	2,9–11; 23,25–27	B
22	Aranyas, H	48°20′N 21°13′E	2,10,16; 5,28–31	B
23	Velebit, HR	44°46′N 14°59′E	11; 22–24	B
24	Durmitor, MNE	43°07′N 19°03′E	15,17; 5,15–17	B
25	Prilep, MK	41°22′N 21°35′E	4,13; 2,14	B
26	Trigrad, BG	41°36′N 24°23′E	1,13; 1,2,3	B
27	Sliven, BG	42°40′N 26°16′E	2,14; 4,5	B
28	Voşlobeni, RO	46°41′N 25°37′E	1,2; 5,16,18	B
29	Baile Herculane, RO	44°52′N 22°25′E	5; 19	B
30	Petreştii de Jos, RO	46°34′N 23°40′E	1,12,19; 5,20,21	B

**Table 2:** Summary statistics of *Polyommatus coridon* population groups for the two sequenced mtDNA gene fragments of COI and CR.

	Haplotype group	n loc	n ind	n hap	Hd	π	r	R^2^	*θ*
COI	A	13	64	20	0.9196	0.00309	0.0555	0.0773	0.00415
	B	8	40	6	0.4949	0.00122	0.1044	0.0719	0.00231
	C	8	40	10	0.7936	0.00364	0.0181	0.1082	0.00396
	D	1	5	2	0.6000	0.00084	0.4000	0.3000	0.00064
	Total	30	149	38	0.9346	0.00868	0.0102	0.0995	0.00805
CR	A	13	63	34	0.9647	0.00821	0.0130	0.0852	0.01030
	B	8	39	20	0.9487	0.00832	0.0086	0.1121	0.00893
	C	8	40	18	0.9423	0.00579	0.0312	0.0951	0.00719
	D	1	5	4	0.9000	0.00180	0.3600	0.2500	0.00173
	Total	30	147	78	0.9858	0.01262	0.0044	0.0806	0.01478
